# O.6.3-3 Gender, ethnicity, and socioeconomic status: intersectoral factors influencing adolescent girls’ participation in sport

**DOI:** 10.1093/eurpub/ckad133.283

**Published:** 2023-09-11

**Authors:** Cecilie Ljungmann, Julie Hellesøe Christensen, Helene Rald Johnsen, Charlotte Demant Klinker, Charlotte Pawlowski

**Affiliations:** Health Promotion Research, Copenhagen University Hospital - Steno Diabetes Center Copenhagen, Denmark; Research Unit for Active Living, Department of Sports Science and Clinical Biomechanics, University of Southern Denmark; cecilie.karen.ljungmann@regionh.dk; Health Promotion Research, Copenhagen University Hospital - Steno Diabetes Center Copenhagen, Denmark; Health Promotion Research, Copenhagen University Hospital - Steno Diabetes Center Copenhagen, Denmark; Health Promotion Research, Copenhagen University Hospital - Steno Diabetes Center Copenhagen, Denmark; Research Unit for Active Living, Department of Sports Science and Clinical Biomechanics, University of Southern Denmark; cecilie.karen.ljungmann@regionh.dk

## Abstract

**Purpose:**

Adolescent girls’ sports participation are low, particularly among females living in low socioeconomic status (SES) neighbourhoods. Inequalities accumulate at the intersection of gender, ethnicity, and socioeconomic status. Understanding this inequity is essential to support better female opportunities in sports participation to reduce inequalities in health. Therefore, the aim of the current study was to investigate how gender, ethnicity, and socioeconomic status influence girls’ participation in sport.

**Methods:**

Eleven focus groups were conducted from July-October 2021 with in total 44 adolescent girls living in low SES neighbourhoods. Ten had Danish ethnic origin and 34 had other ethnic minority background of whom 18 were born in Denmark with parents born in non-western countries. 21 of the girls were wearing veil as a symbol of their religious background. The inclusion criteria were non-participation in organised sport in their leisure time. The girls were recruited through purposive sampling via schools placed in five different low SES neighbourhoods. Drawing on an intersectoral framework as a tool to understand the complex interaction between gender, ethnicity, and socioeconomic status, a deductive thematic analysis was conducted from verbatim transcripts using NVivo.

**Results:**

Capturing the girls’ own voices, the analyses identified three overarching themes; 1) The little voice inside, 2) Sports are not for girls, 3) Can you do sport with veil? Findings revealed that the barriers for this group of girls had more complex intertwined layers, where gender, ethnicity and socioeconomic status were accumulated. The analyse indicates that gendered societal expectations and stereotypes about femininity, along with cultural and religious norms and socioeconomic factors, act as a barrier for girls to participate in sport.

**Conclusions:**

The results provide insight into intersectional factors that influence sports participation among adolescent girls living in low SES neighbourhoods. The findings among this underrepresented group indicate that gender, ethnicity, and socioeconomic status should be considered to ensure equal opportunities to sports participation. The findings will be useful to shed light on how interventions should be designed to promote sports participation among adolescent girls in low SES neighbourhoods and thus help promote equity.

